# Extraordinary centromeres: differences in the meiotic chromosomes of two rock lizards species *Darevskia portschinskii* and *Darevskia raddei*

**DOI:** 10.7717/peerj.6360

**Published:** 2019-01-30

**Authors:** Victor Spangenberg, Marine Arakelyan, Eduard Galoyan, Mark Pankin, Ruzanna Petrosyan, Ilona Stepanyan, Tatiana Grishaeva, Felix Danielyan, Oxana Kolomiets

**Affiliations:** 1 Vavilov Institute of General Genetics, Moscow, Russian Federation; 2 Department of Zoology, Yerevan State University, Yerevan, Armenia; 3 Zoological Museum, Moscow State University, Moscow, Russia; 4 Scientific Center of Zoology and Hydroecology, Yerevan, Armenia

**Keywords:** *Darevskia* lizards, Synaptonemal complex, Dicentric chromosomes, Reticulate evolution, Neocentromere, Meiosis

## Abstract

According to the synthesis of 30 years of multidisciplinary studies, parthenogenetic species of rock lizards of genus *Darevskia* were formed as a result of different combination patterns of interspecific hybridization of the four bisexual parental species: *Darevskia raddei*, *D. mixta*, *D. valentini*, and *D. portschinskii*. In particular, *D. portschinskii* and *D. raddei* are considered as the parental species for the parthenogenetic species *D. rostombekowi*. Here for the first time, we present the result of comparative immunocytochemical study of primary spermatocyte nuclei spreads from the leptotene to diplotene stages of meiotic prophase I in two species: *D. portschinskii* and *D. raddei*. We observed similar chromosome lengths for both synaptonemal complex (SC) karyotypes as well as a similar number of crossing over sites. However, unexpected differences in the number and distribution of anti-centromere antibody (ACA) foci were detected in the SC structure of bivalents of the two species. In all examined *D. portschinskii* spermatocyte nuclei, one immunostained centromere focus was detected per SC bivalent. In contrast, in almost every studied *D. raddei* nuclei we identified three to nine SCs with additional immunostained ACA foci per SC bivalent. Thus, the obtained results allow us to identify species-specific karyotype features, previously not been detected using conventional mitotic chromosome analysis. Presumably the additional centromere foci are result of epigenetic chromatin modifications. We assume that this characteristic of the *D. raddei* karyotype could represent useful marker for the future studies of parthenogenetic species hybrid karyotypes related to *D. raddei*.

## Introduction

According to the results of long term fundamental international studies initiated by Darevsky (1958, 1966, 1967, 1992) convincing evidence has been obtained that seven diploid parthenogenetic species of lizards of the *Darevskia* genus have resulted from hybridogenous speciation (Borkin & Darevsky, 1980; Moritz et al., 1992; Murphy et al., 1996, 2000; Fu, 1998; Fu, Murphy & Darevsky, 2000; Freitas et al., 2016; Ryskov et al., 2017). The origin of parthenogenetic species from the hybridization of bisexual species has been confirmed from detailed studies of skin transplantation (Darevsky & Danielyan, 1979; Danielyan, 1987; Korkiya, 1976), allozyme data (Murphy et al., 1996, 2000; Uzzell & Darevsky, 1974, 1975; MacColloch et al., 1995), mitochondrial (Moritz et al., 1992; Fu, 1998; Fu, Murphy & Darevsky, 1997, 2000), and nuclear DNA sequences (Freitas et al., 2016; Ryskov et al., 2017; Khan et al., 1998; Tokarskaya et al., 2001; Grechko et al., 2006; Omelchenko et al., 2016).

The balance hypothesis suggest that there is a narrow range of genetic divergence between parental species within which F1 hybrids have a probability of establishing parthenogenetic form (Murphy et al., 2000; Moritz et al., 1989).

In this study, we performed a detailed analysis of the meiotic prophase I stages of two species: *D. portschinskii* and *D. raddei* which are parental for the parthenogenetic species *D. rostombekowi*. Previous cytogenetic studies of these two species were performed using light microscopy on mitotic metaphase plates (Darevsky & Kupriyanova, 1982; Darevsky, Kupriyanova & Danielyan, 1986) and are sporadic. Here, we represent detailed comparative cytogenetic study of synaptonemal complexes karyotypes (SC karyotypes) using spread preparation and immuno-fluorescent *in situ* hybridization (FISH) technique. This approach provides visualization of meiotic SC bivalents which are three to five times longer than mitotic metaphase chromosomes and makes it possible to discover chromosomal rearrangements that are undetectable at diakinesis and metaphase I (Kalikinskaya et al., 1986). Additional information can also be obtained: precise localization of centromeres, distribution of crossing over sites, and telomere DNA-repeats in the structure of meiotic chromosomes.

## Materials and Methods

Four adult animals were captured and examined in May 2017 and two in September 2017 and were deposited in the research collection of the Zoological Museum of Lomonosov Moscow State University (ZMMU). One male *D. raddei* (Zuar population, ZMMU R-15598, specimen VS0029) collected by E.A. Galoyan and V.E. Spangenberg in May 2017, one male *D. raddei* (Zuar population, ZMMU R-15599, specimen VS0039) collected by M.S. Arakelyan and V.E. Spangenberg in September 2017) and two males *D. portchinskii* (Zuar population, ZMMU R-15600, specimen VS0028, ZMMU R-15600, specimen VS0050) collected by M.S. Arakelyan, E.A. Galoyan, and V.E. Spangenberg in May and September 2017, respectively. The manipulations of the animals followed international rules of the Manual on Humane Use of Animals in Biomedical Research and the rules of the Ethics Committee for Animal Research of the Vavilov Institute of General Genetics (protocol No. 3 from November 10, 2016).

Spread SC preparations were prepared and fixed using the technique of Navarro et al. (1981). Poly-l-lysine-coated slides were used for all immunofluorescence studies. The slides were washed with phosphate-buffered saline (PBS) and incubated overnight at 4 °C with primary antibodies diluted in antibody dilution buffer (ADB: 3% bovine serum albumin, 0.05% Triton X–100 in PBS).

Synaptonemal complexes were detected by rabbit polyclonal antibodies to the SC and axial element protein SYCP3 (1:250; Abcam, Cambridge, UK), centromeres were detected by anti-kinetochore proteins antibodies ACA (1:500; Antibodies Incorporated, Davis, CA, USA). The late recombination nodules (sites of crossing over) were detected using mouse monoclonal antibodies to the DNA mismatch repair protein — MLH1 (1:250; Abcam, Cambridge, UK). After washing, we used the secondary antibodies diluted in ADB: goat anti mouse immunoglobulin G (IgG), Alexa Fluor 555 (1:500; Abcam, Cambridge, UK), Rhodamine-conjugated chicken anti-rabbit IgG (1:400; Santa Cruz Biotechnology, Dallas, TX, USA), FITC-conjugated goat anti-rabbit IgG (1:500; Jackson ImmunoResearch, West Grove, PA, USA), goat anti-rabbit Alexa Fluor 488 (1:500; Invitrogen, Carlsbad, CA, USA), goat anti-human Alexa Fluor 546 (1:500; Invitrogen, Carlsbad, CA, USA). Secondary antibody incubations were performed in a humid chamber at 37 °C for 2 h. Mitotic chromosomes were prepared from bone marrow and spleen following Ford and Hamerton with modifications and fixed in an ice-cold acetic acid–methanol solution (1:3) (Ford & Hamerton, 1956). Telomere FISH probe (Telomere PNA FISH Kit/FITC, Dako, K5325) was used according to the manufacturer protocol.

The slides were examined using an AxioImager D1 microscope (Carl Zeiss, Oberkochen, Germany) equipped with an Axiocam HRm CCD camera (Carl Zeiss, Oberkochen, Germany), Carl Zeiss filter sets (FS01, FS38HE, and FS43HE) and image-processing AxioVision Release 4.6.3. software (Carl Zeiss, Oberkochen, Germany). All preparations were mounted in Vectashield antifade mounting medium with DAPI (Vector Laboratories, Burlingame, CA, USA). CENP proteins were compared by alignment (COBALT software program, http://www.ncbi.nlm.nih.gov/tools/cobalt/cobalt.cgi?CMD=Web).

Prophase I stages were determined by the analysis of the combination of basic morphological criteria used in studies of meiotic cells (Zickler & Kleckner, 1999; Bogdanov & Kolomiets, 2007). The rock lizards-specific features of the prophase I stages were described before (Spangenberg et al., 2017). Early presynaptic stages criteria for leptotene: multiple fragments of unpaired axial elements, and for the zygotene: long partially synapsed axial elements, «bouquet» formation (telomere clustering at zygotene), no signs of desynapsis in telomere regions, no MLH1-protein foci. Mid-prophase I stage (pachytene) criteria: complete homologous chromosome synapsis, non-fragmented lateral elements of SCs, MLH1-protein foci. Postsynaptic stage (diplotene) criteria: signs of SCs disassembly (lateral elements desynapsis start in peritelomeric or interstitional regions, elongation and fragmentation), MLH1-protein foci maintenance.

## Results

### Early stages of meiotic prophase I in *D. portschinskii* and *D. raddei*: leptotene and zygotene

**Figure 1:**
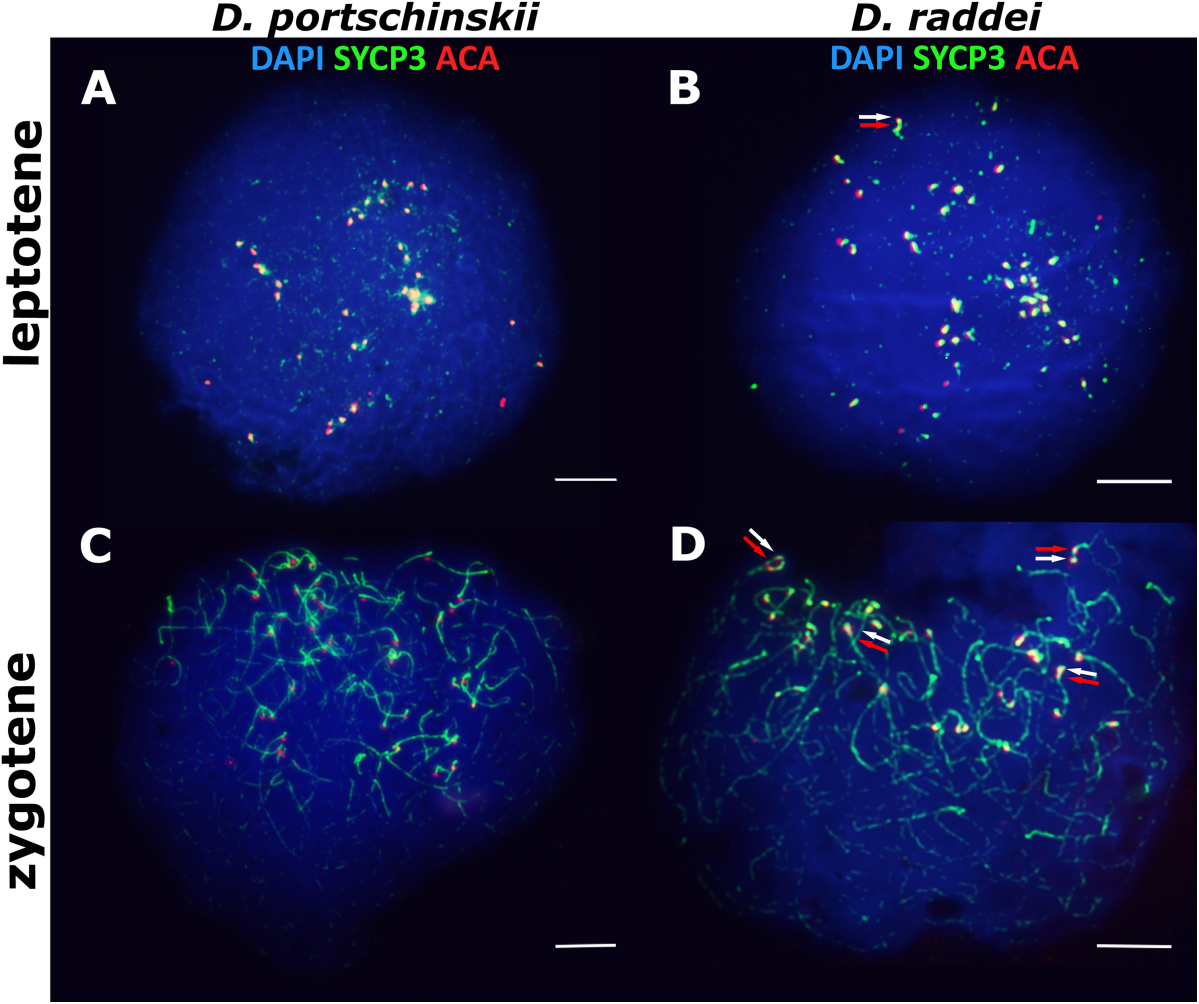
The spread nuclei of the leptotene–zygotene spermatocytes. *D. portschinskii* (A and C) and *D. raddei* (B and D). Synaptonemal complexes (SCs) immunostained with antibodies against the SYCP3 protein (green), centromeres - with anti-kinetochore ACA antibodies (red), chromatin stained with DAPI (blue). (A and C) *D. portschinskii*, on each of the 19 SCs only one centromere signal is visible. (B and D) *D. raddei*, additional anti-kinetochore proteins antibodies signals are indicated with arrows. Bar = five μm.

In both species *D. portschinskii* and *D. raddei*, leptotene begins with the formation of axial structures, which resemble dotted lines (Figs. 1A and 1B) until the early zygotene (Figs. 1C and 1D). Assembly of chromosome axial structures from the fragments was often observed together with the beginning of homologous synapsis (early “bouquet” formation) in different zones of one nucleus (Figs. 1C and 1D). Immunostaining of kinetochore proteins revealed progressive clustering of the centromeres of all 38 acrocentric chromosomes from the leptotene to early zygotene stages in both species (Figs. 1C and 1D).

**Figure 2:**
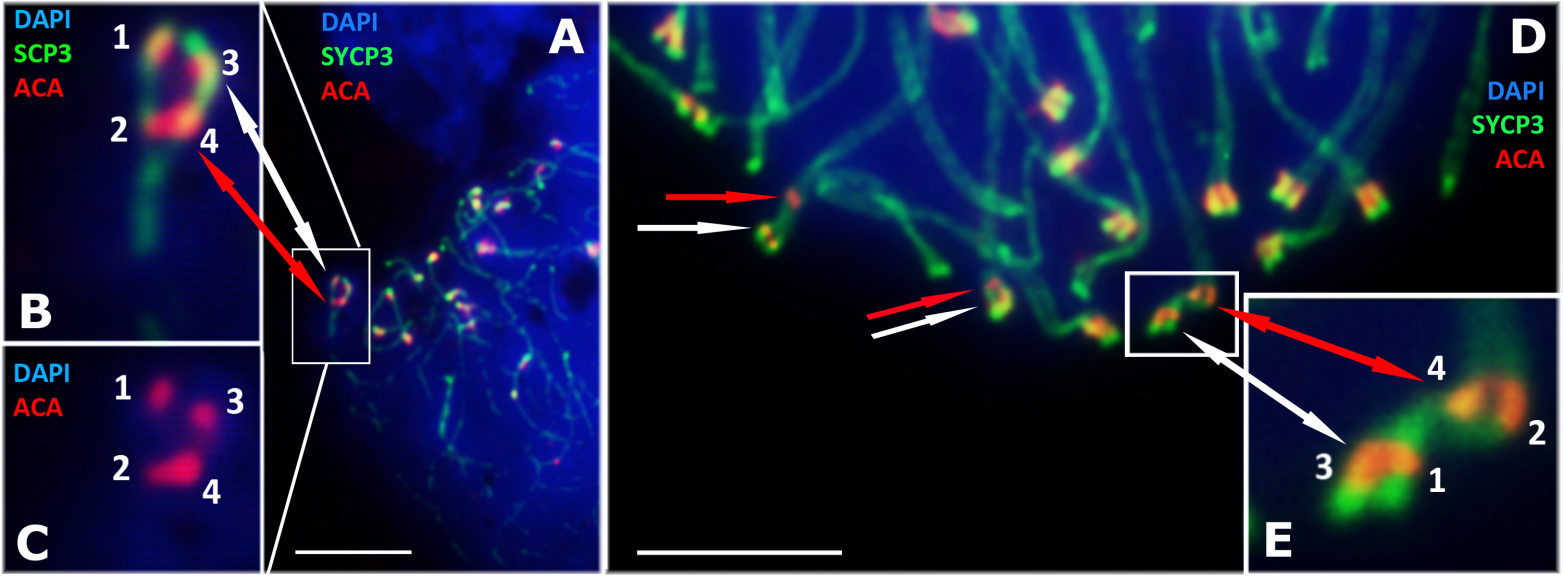
*D. raddei* synaptonemal complexes, zygotene stage. SCs immunostained with antibodies against the SYCP3 protein (green), centromeres with anti-kinetochore antibodies ACA (red). Chromatin stained with DAPI (blue). (A) Early zygotene. SC-bivalent with incomplete synapsis of pericentromeric region. (B, C) Enlarged fragments of A. (D) Late zygotene. Additional ACA-signals indicated with red arrows. (E) Enlarged fragment of D, demonstrate additional ACA foci on each axial element prior to synapsis. Bar = five μm.

Our detailed study of early prophase I stages (preceding the pachytene) revealed previously unknown phenomenon specific for *D. raddei*—additional foci of anti-kinetochore proteins antibodies ACA (Figs. 1B and 1D). Additional ACA foci were detected on the still unsynapsed axial elements of *D. raddei* homologous chromosomes at zygotene stage (Figs. 2A and 2D). Thus, four ACA foci were visible on several SC bivalents during the synapsis, with two on each axial element (Figs. 2B, 2C and 2E). Totally in 26 of 30 leptotene–zygotene nuclei studied one to four additional centromere signals were detected in both *D. raddei* individuals. Fragmented axial cores of chromosomes at the presynaptic stages (before the pachytene) did not allow us to study actual number and distribution of additional ACA foci among the meiotic chromosomes. The next meiotic prophase I stage, pachytene, was analyzed in details due to complete SCs assembly and applicability for the distinct chromosomes identification.

### Late stages of meiotic prophase I in *D. portschinskii* and *D. raddei*: pachytene and diplotene

**Figure 3:**
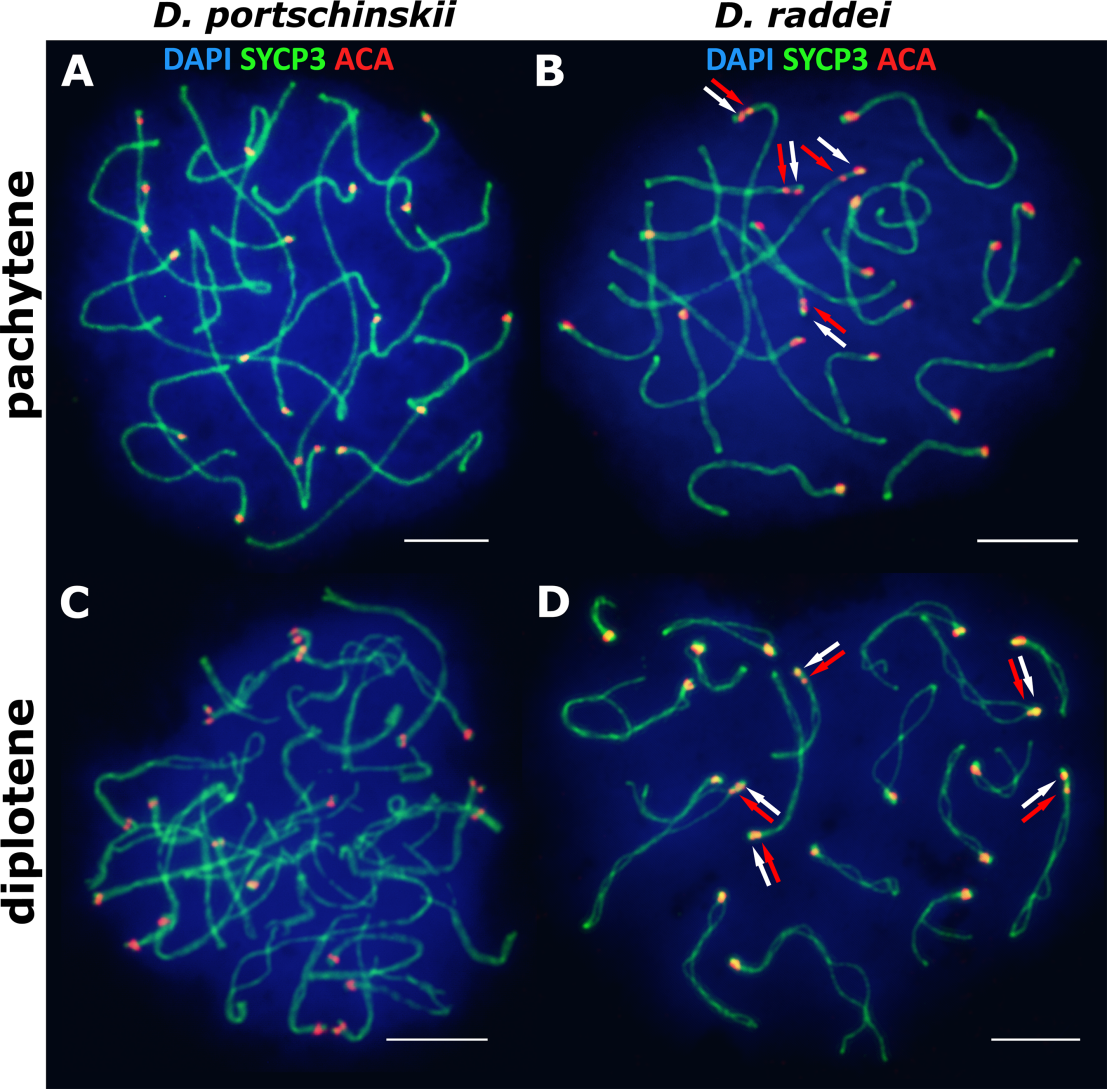
The spread nuclei of the pachytene–diplotene spermatocytes. SCs immunostained with antibodies against the SYCP3 protein (green), centromeres - with anti-kinetochore antibodies ACA (red). Chromatin stained with DAPI (blue). (A and C) *D. portschinskii*. On each of the 19 SCs only one centromere signal is visible. (B and D) *D. raddei*. Additional anti-kinetochore proteins antibodies signals are indicated with arrows. Bar = five μm.

The pachytene stage of both species studied (*D. portschinskii* and *D. raddei*) is characterized by complete synapsis of all 19 acrocentric SC bivalents (18 autosomal bivalents and the ZZ sex bivalent) (Figs. 3A and 3B).

In *D. portschinskii* pachytene nuclei single ACA focus was detected at one end of each of the 19 SCs (78 nuclei studied) (Fig. 3A). However, in *D. raddei* apart from to the usual 19 ACA foci on the SC ends we detected additional ACA foci on the SC bivalents (Fig. 3B). These signals were located closely in the SC structure at a distance of 0.27–1.39 μm, on average 0.62 ± 0.21 (mean ± SD, 54 nuclei studied) and demonstrated similar or slightly different signal intensities.

**Figure 4:**
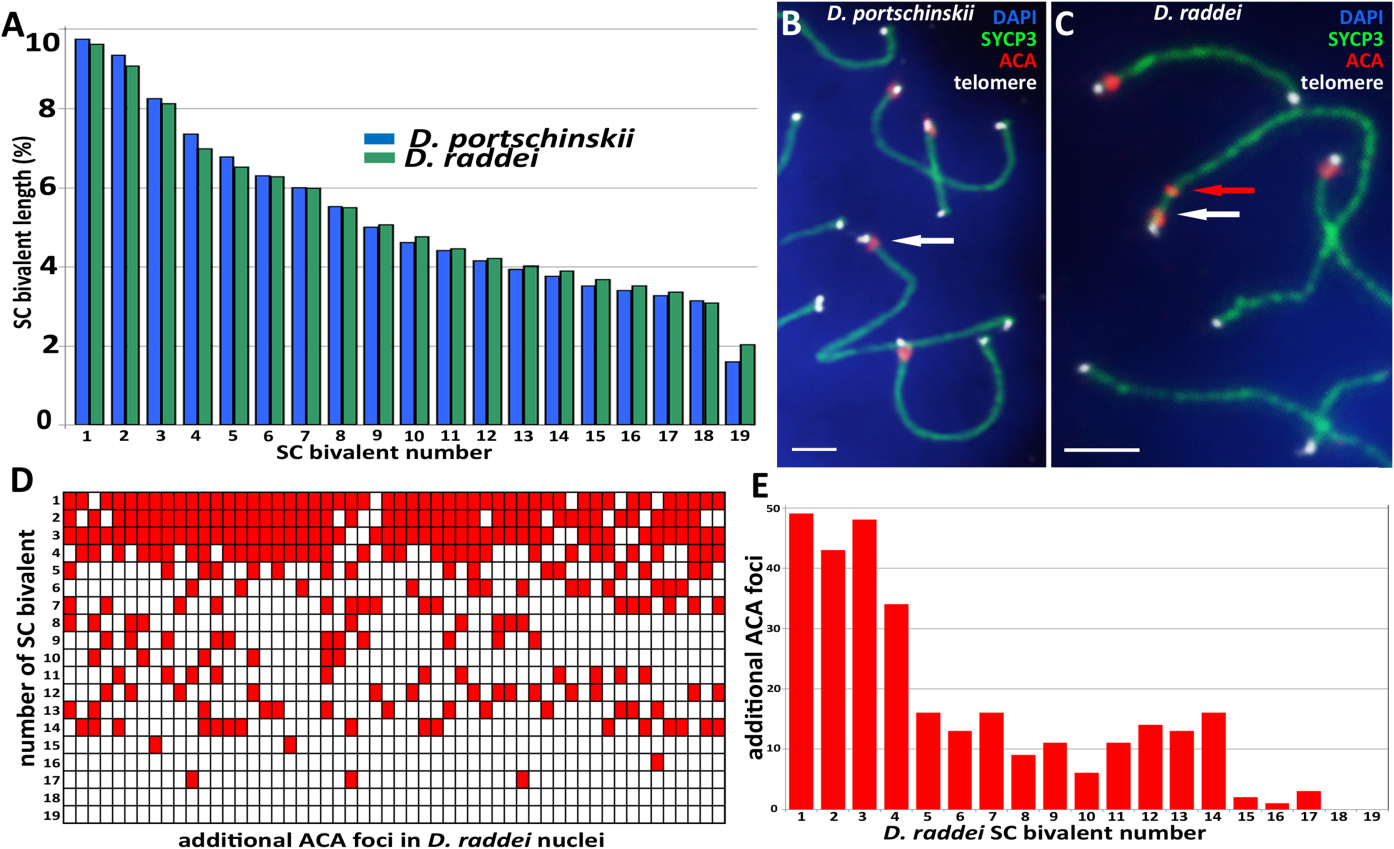
A comparison idiogram of average SC lengths of two species, distribution of additional ACA foci and immuno-FISH with telomere probes. (A) A comparison idiogram of average SC lengths of two species: *D. portschinskii* (blue, 78 pachytene nuclei) and *D. raddei* (green, 54 pachytene nuclei). (B and C) Fragments of SC preparations *D. portschinskii* and *D. raddei*, immuno-FISH. SCs immunostained with antibodies against the SYCP3 protein (green), centromeres with anti-kinetochore antibodies ACA (red), telomeres labelled by PNA telomere FISH probe (white), chromatin stained with DAPI (blue). (B) Single ACA focus on each of *D. portschinskii* SC bivalents; (C) two ACA foci in *D. raddei* SC is (red and white arrows). Telomere FISH signals (white) are located at the ends of SC bivalents in both species and were not detected in the area of the additional ACA focus in *D. raddei*. (D and E) Distribution of additional ACA-foci on the 19 SC-bivalents of *D. raddei* in 54 spermatocyte nuclei studied. Bar = two μm.

Pachytene stage was the most representative for the precise chromosome length measurements, other prophase I stages are inapplicable for this study. Nevertheless SC karyotyping revealed minor differences in the length of medium-sized SC bivalents in both species (Fig. 4A) making the identification of distinct dicentric (Fig. 4C) chromosomes more challenging.

We performed the detailed analysis of 54 pachytene nuclei of the six preparations from two *D. raddei* animals. We selected immunostained SC-karyotypes without bivalent overlapping and used relative chromosomes length in order to minimize possible influence of the different spreading conditions between nuclei studied. Additional ACA foci were detected at chromosomes 1–17 with enrichment on chromosomes 1–4 (1—90.7%; 2—79.6%; 3—88.9%; 4—63.0% of all 54 nuclei studied), medium occurrence on chromosomes 5–14 (5—29.6%; 6—24.1%; 7—29.6%; 8—16.7%; 9—20.4%; 10—11.1%; 11—20.4%; 12—25.9%; 13—24.1%; 14—29.6% of 54 nuclei studied), sporadic occurrence on chromosomes 15–17 (15—3.7%; 16—1.9%; 17—5.6% of 54 nuclei studied) and not been detected on the chromosomes 18 and 19 (Figs. 4D and 4E).

In total, additional ACA foci were detected in 198 out of the 211 *D. raddei* primary spermatocyte nuclei studied (leptotene–diplotene stages).

### FISH with telomere probes on pachytene chromosomes of *D. portschinskii* and *D. raddei*

Fluorescent *in situ* hybridization with telomere probes (Figs. 4B and 4C) revealed a standard distribution of telomere repeats in the SC karyotypes of *D. portschinskii and D. raddei*, with no interstitial signals (Rovatsos et al., 2015) detected in the pachytene SC bivalents (25 nuclei for each species studied). In particular, we studied the SC regions of additional ACA foci in *D. raddei* SC bivalents, and no one telomere FISH-signal was detected at any of these locations in all nuclei studied (Fig. 4C).

### Immunodetection of crossing over sites in *D. portschinskii* and *D. raddei* spermatocytes I

**Figure 5:**
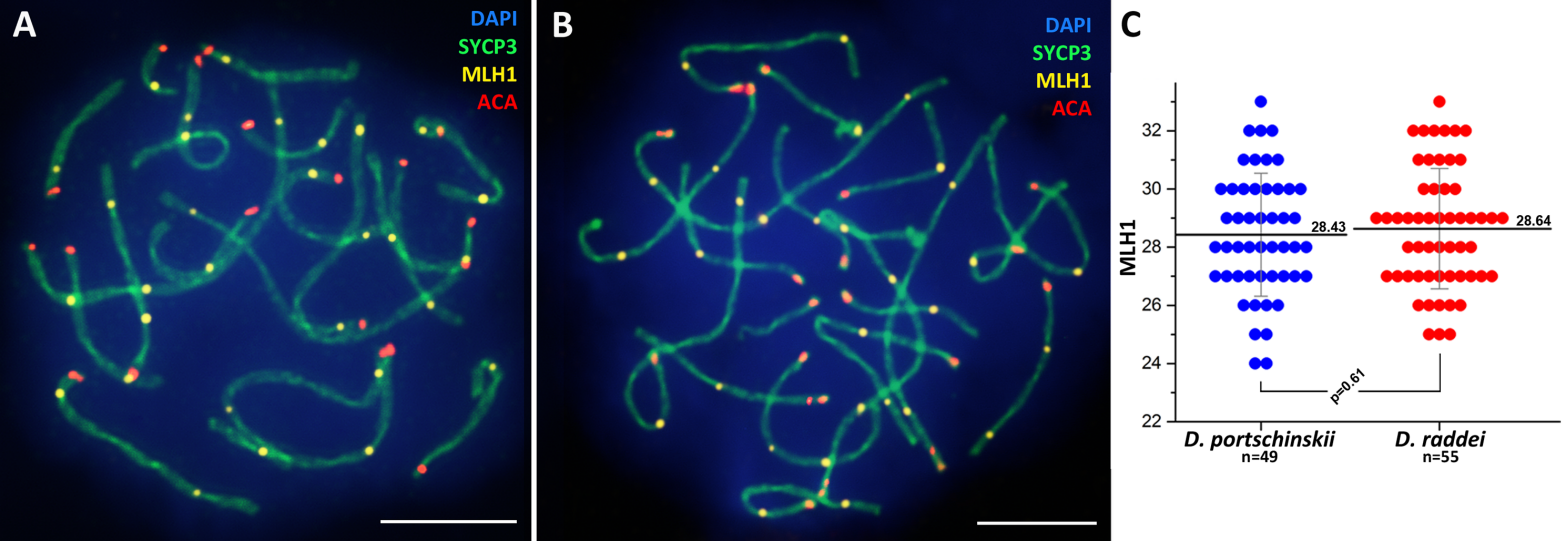
Immunodetection of crossing over sites in *D. portschinskii* and *D. raddei*. SC spreads of *D. portschinskii* (A) and *D. raddei* (B) immunostained with antibodies against the SYCP3 protein (green)—lateral elements of meiotic chromosomes, anti-kinetochore ACA antibodies (red). Sites of crossing over immunostained with antibodies against mismatch repair protein MLH1 (yellow). (C) Number of crossing over sites (MLH1 foci) per spermatocyte nucleus (mean ± SD) in *D. portschinskii* and *D. raddei*. Bar = five μm.

Immunolocalization of the MLH1 protein (late recombination nodules marker, prospective chiasmata) on pachytene stage in the SC preparations was performed for both species (Figs. 5A and 5B). The average number of crossing over sites (MLH1 foci) was 28.43 ± 2.11 (mean ± SD) in *D. portschinskii* (Fig. 5C) and 28.64 ± 2.07 (mean ± SD) in *D. raddei* (Fig. 5C). We did not detect MLH1 foci in the SC regions between the two ACA foci in all 55 immunostained *D. raddei* spermatocyte I nuclei (Fig. 5B).

## Discussion

### Synaptonemal complex processing and number of crossing over sites during prophase I in *D. portschinskii* and *D. raddei*

In general, the characteristics of the stages of meiotic prophase I were similar between species (Figs. 1 and 3), and were comparable to the results of our previous study of two other bisexual species, *D. raddei nairensis* and *D. valentini* (Spangenberg et al., 2017). For example, the absence of a classical leptotene stage with long threads (presynaptic stage with completely formed chromosome axial elements) is a common feature among all four rock lizards species studied by us to date. Furthermore, early zygotene, organization of “bouquet” formation, progression of axial element synapsis during zygotene and SC assembly at pachytene were similar in both species (Figs. 1C, 1D, 3A and 3B). No significant difference in numbers of crossing over sites was detected between *D. portschinskii* and *D. raddei* males (Fig. 5C) in the studied population.

### Extraordinary difference of distribution of ACA foci in the SC bivalents in *D. portschinskii* and *D. raddei*

As visible in our results, the SC karyotypes of the two species display a striking difference in relation to the immunostaining of kinetochore proteins. Two ACA foci can be observed in the structure of SC bivalents 1–17 in primary spermatocyte nuclei of *D. raddei* (Figs. 4D and 4E), one near the telomere, similar to the acrocentric organization of SCs in *D. portschinskii*, and the second located at some distance along the SC axis (Fig. 4C). Spreading technique allow us to study meiotic chromosomes in detail (Fig. 4C) and to detect additional ACA foci. This phenomenon has not been previously described in mitotic chromosomes due to high levels of chromatin compaction as well as in previous studies of the meiotic chromosome structure of rock lizards using light microscopy (Darevsky & Kupriyanova, 1982; Darevsky, Kupriyanova & Danielyan, 1986).

Immunodetection of additional ACA foci in *D. raddei* during the leptotene and zygotene stages indicates the presence of two ACA foci in both homologous chromosomes prior to synapsis (Figs. 1B, 1D, 2B, 2C and 2E).

The differences in additional ACA foci number and distribution were detected in the nuclei from one sample preparation as well as between preparation slides derived from different *D. raddei* animals. We suppose that this result can be explained by the fact that closely located double ACA foci often cannot be distinguished from enlarged (or elliptical) single focus due to limitations of fluorescence microscopy as well as because the nearest chromosome lengths are very close in this species (Fig. 4A). On the other hand, the different chromosome numbers with additional ACA foci from the one sample could indicate a high level of instability of centromere protein distribution in the pericentromeric regions in *D. raddei*.

Additional ACA foci we detected in SC-karyotype of *D. raddei* males leads us to assume that the formation of the additional foci occurred not via inversions or duplication but via an epigenetic mechanism in these species. This is supported by the fact that we did not observe any disruption of synapsis or inversion loops in the pericentomeric regions of SC bivalents with additional ACA foci in all 198 nuclei studied.

In recent years, studies in mammals have reported that neocentromeres can be formed in intact chromosomes (without rearrangements), which can functionally replace the native centromeres (Rocchi et al., 2012). De novo centromere formation occurs by an epigenetic sequence-independent mechanism involving the deposition of a centromere-specific histone H3 variant, CENP-A (Allshire & Karpen, 2008; Black & Cleveland, 2011; Perpelescu & Fukagawa, 2011). A study of artificial neocentromere formation in chickens demonstrated that while they can be formed in any region of the chromosome, the most likely location of neocentromere formation is close to the native centromere. The authors suggested that this is due to the potential enrichment of epigenetic marks in the zone of the native centromere (Shang et al., 2013; Zhao et al., 2017). According to studies of mitosis in different species, the formation of dicentric chromosomes (chromosomes with two active centromeres) may lead to breakage or loss of such chromosomes during the process of cell division and, consequently, cell death (Stimpson, Matheny & Sullivan, 2012; Lopez et al., 2015). However, dicentric chromosomes can be inherited if one of the centromeres is inactivated without altering the DNA sequence (Earnshaw & Migeon, 1985; Valente, Silva & Jansen, 2012). Furthermore, immunocytochemical markers allow the identification of which of the two centromeres is active (Voullaire et al., 1993; Warburton et al., 1997). However, the molecular basis for centromere inactivation is not well understood (Stimpson, Matheny & Sullivan, 2012).

Our results do not allow the status of additional ACA foci to be determined, specifically whether they are associated with active or inactivated centromeres. However, the number of nuclei with clear additional ACA foci, identified in two *D. raddei* males, indicates that this phenomenon is not random. Moreover, numerous mature elongated spermatids with normal morphology were found in preparations from both *D. raddei* males in the current study. The successful formation of mature germ cells by *D. raddei* males indicates that the peculiarities of centromere organization described above does not affect their fertility.

The formation of double centromeres in the structure of SCs has been previously reported for *Danio rerio* fish (Moens, 2006). In this study, three to four double centromeres were detected in the nuclei of primary spermatocytes, which the author characterized as being misaligned centromeres. The author also pointed out that such double centromeres are not necessarily found on the same chromosomes, judging by the length and centromere position (Moens, 2006). This pattern correlates well with the distribution of the double ACA foci observed for *D. raddei* in the current study. However, in the early zygotene nuclei of *D. raddei* males, we were able to detect two ACA foci on the yet unpaired axial elements of chromosomes. Furthermore, four ACA foci were visible by the zygotene stage, with two on each axial element (Figs. 2B, 2C and 2E). This indicates the presence of two ACA antibody binding sites on each axial element but not incomplete alignment of homologous chromosomes.

It should be noted that we used similar antibodies (ACA) to those used by other studies (ACA, CREST) (Brenner et al., 1981; Oppedisano et al., 2002; Chambon et al., 2013). The applicability of these antibodies for the detection of centromeres in a wide range of vertebrate species has long been established. In addition, numerous studies in the literature have discussed the specific characteristics of immunostaining using these antibodies during different phases of the cell life cycle (Brenner et al., 1981; Chambon et al., 2013). Our comparative studies of CENP-A and CENP-C proteins by alignment of amino acids sequences confirmed our negative immunostaining experiments with the commercially available CENP-antibodies. Poor similarity was detected between reptilian proteins and the human immunogen polypeptides used in commercial antibodies.

Further studies using monoclonal antibodies against specific proteins are needed in order to distinguish active and inactivated centromeres, in addition to FISH with pericentromeric satellite DNA. An interesting finding was the detection of additional ACA foci in SC preparations of the most closely related species, *D. raddei nairensis* (in supplemental data: see ‘Data Availability’ section). *D. raddei* and *D. raddei nairensis* are considered recently divergent species, and are actively investigated as a model system for the study of speciation mechanisms (Omelchenko et al., 2016).

## Conclusions

Our comparative immunomorphological study revealed that karyotypes of both species were found to be very similar with regard to chromosome length, with similar features related to passage through the stages of meiotic prophase I and the number of crossing over sites.

Nevertheless, we were able to detect striking difference in the number and distribution of centromere proteins foci of these species: the formation of additional ACA foci in the structure of several SC bivalents in the spermatocyte nuclei of *D. raddei* but not in *D. portschinskii*. It is important to note that additional ACA foci were detected during all stages of meiotic prophase I, including the presynaptic stages, on the yet unpaired axial elements of homologous chromosomes. Thus, we observed SC during the assembly with four ACA foci at the zygotene stage, two on each axial element. This indicates the presence of two ACA antibodies binding sites in the structure of each homologous chromosomes, thus eliminating the idea of incomplete alignment of homologous chromosomes during synapsis or desynapsis.

The additional ACA foci observed in the SC structure appeared to result from epigenetic transformations in the chromatin structure of *D. raddei* males, and are not related to chromosomal aberrations. In any case, we did not observed the formation of inversion loops in SC fragments between two ACA foci in any of the 198 studied nuclei (leptotene–diplotene).

Further research is needed to determine the possible interpretation of the extraordinary chromosome organization observed in *D. raddei* in relation to the active and surprisingly efficient interspecific hybridization in rock lizards. In particular, a detailed study of the epigenetic chromatin modifications of hybrid animals is required. We assume that this characteristic of the *D. raddei* karyotype could represent useful marker for our future study of diploid parthenogenetic species *D. rostombekowi* karyotype as well as for other unisexual species related to *D. raddei*.

## Additional Information and Declarations

### Data Availability

The following information was supplied regarding data availability: Spangenberg, Victor (2018): RAW DATA ROCK LIZARDS 3 species. figshare. Dataset. https://doi.org/10.6084/m9.figshare.7466750.v1. Victor Spangenberg. (2018). raw images 3 species chromosomes. Zenodo. http://doi.org/10.5281/zenodo.2232368.
